# Invertases in Phytophthora infestans Localize to Haustoria and Are Programmed for Infection-Specific Expression

**DOI:** 10.1128/mBio.01251-20

**Published:** 2020-10-13

**Authors:** Meenakshi S. Kagda, Domingo Martínez-Soto, Audrey M. V. Ah-Fong, Howard S. Judelson

**Affiliations:** a Department of Microbiology and Plant Pathology, University of California, Riverside, California, USA; MPI for Terrestrial Microbiology

**Keywords:** metabolism, nutrition, oomycetes, plant pathogens, transcriptional regulation

## Abstract

The oomycete Phytophthora infestans, the causal agent of potato and tomato blight, expresses two extracellular invertases. Unlike typical fungal invertases, the *P. infestans* genes are not sucrose induced or glucose repressed but instead appear to be under developmental control. Transcript levels of both genes were very low in mycelia harvested from artificial medium but high in preinfection stages (sporangia, zoospores, and germinated cysts), high during biotrophic growth in leaves and tubers, and low during necrotrophy. Genome-wide analyses of metabolic enzymes and effectors indicated that this expression profile was fairly unusual, matched only by a few other enzymes, such as carbonic anhydrases and a few RXLR effectors. Genes for other metabolic enzymes were typically downregulated in the preinfection stages. Overall metabolic gene expression during the necrotrophic stage of infection clustered with artificial medium, while the biotrophic phase formed a separate cluster. Confocal microscopy of transformants expressing green fluorescent protein (GFP) fusions indicated that invertase protein resided primarily in haustoria during infection. This localization was not attributable to haustorium-specific promoter activity. Instead, the N-terminal regions of proteins containing signal peptides were sufficient to deliver proteins to haustoria. Invertase expression during leaf infection was linked to a decline in apoplastic sucrose, consistent with a role of the enzymes in plant pathogenesis. This was also suggested by the discovery that invertase genes occur across multiple orders of oomycetes but not in most animal pathogens or a mycoparasite.

## Abstract (precis)

Oomycetes cause hundreds of diseases in economically and environmentally significant plants. How these microbes acquire host nutrients is not well understood. Many oomycetes insert specialized hyphae called haustoria into plant cells, but unlike their fungal counterparts, a role in nutrition has remained unproven. The discovery that *Phytophthora* invertases localize to haustoria provides the first strong evidence that these structures participate in feeding. Since regions of proteins containing signal peptides targeted proteins to the haustorium-plant interface, haustoria appear to be the primary machinery for secreting proteins during biotrophic pathogenesis. Although oomycete invertases were acquired laterally from fungi, their expression patterns have adapted to the *Phytophthora* lifestyle by abandoning substrate-level regulation in favor of developmental control, allowing the enzymes to be produced in anticipation of plant colonization. This study highlights how a widely distributed hydrolytic enzyme has evolved new behaviors in oomycetes.

## INTRODUCTION

A primary goal of pathogens is to acquire host nutrients to support growth and reproduction. Important to plants and pathogens is the disaccharide sucrose. During normal plant growth, sucrose is made from photosynthate in green source tissues, exported to the apoplast, loaded into phloem, and delivered to sink tissues such as roots, tubers, and fruits (1). In these sinks, sucrose enters plant cells through sucrose transporters or is taken up by hexose transporters after hydrolysis into glucose and fructose by cell wall invertases (β-fructofuranosidase; EC 3.2.1.26). Interestingly, these plant invertases are activated during pathogen attack, increasing the hexose/sucrose ratio in the apoplast (2, 3). While this may stimulate the plant defense response, it may also nourish apoplast-adapted pathogens, which include many fungi, bacteria, and oomycetes.

Many of these microbes encode their own invertases as well as sugar transporters, which cause the pathogen to acquire more sugar by becoming a sucrose sink (4, 5). For example, bacterial invertases, typically termed sucrose hydrolases, are considered pathogenicity factors (6). The Uromyces fabae invertase has been shown to generate hexoses that are taken up by that fungus’ own transporter (7). Oomycetes such as *Phytophthora* spp. also express invertases. These have been little studied but are thought to have been acquired from fungi by horizontal gene transfer (8).

The present study focused on invertases in the potato and tomato blight pathogen Phytophthora infestans. Infections by this hemibiotrophic oomycete usually begin when asexual sporangia release zoospores, which later encyst on host tissues and extend germ tubes that enter the host (9). Biotrophic growth then typically proceeds for several days, during which time the pathogen feeds from the apoplast using intercellular hyphae. These also produce haustoria, which establish membrane interfaces with plant cells. The transition between biotrophy and necrotrophy is associated with a decline in haustorium formation and alterations in metabolic enzyme production (10, 11). For example, only late in infection does *P. infestans* produce much enzyme for degrading nutrients, such as starch, that are normally sequestered within plant cells.

Unlike their fungal counterparts, oomycete haustoria have not been proved to play a role in nutrient uptake. Haustoria are known to deliver effectors into plants using conventional and nonconventional (brefeldin A-insensitive) secretion pathways (12). The RXLR motif, the signature of the major class of *Phytophthora* effectors, has been proposed to direct proteins to the plant-haustorium interface, although the role of that sequence is controversial (13).

How invertases are regulated in any pathogen, including oomycetes, is not well understood. Studies in nonpathogenic yeasts and filamentous fungi have shown that invertase transcription is usually induced by sucrose (14, 15). Glucose often inhibits expression, through either the carbon catabolite repression pathway or translational control (16).

In this study, we focused on the expression pattern and localization of invertases in *P. infestans.* Unlike most fungal invertases studied to date, the *P. infestans* genes do not appear to be sucrose induced but are under developmental control. Expression begins in spores, continues during biotrophic growth, and is suppressed during necrotrophy and growth on artificial medium. The invertases localize to haustoria, providing the first strong evidence that oomycete haustoria play a role in acquiring nutrients. We also propose that a signal peptide may be the only sequence needed to target proteins to haustoria, which helps to explain its role as an organ for polarized secretion.

## RESULTS

### Apoplastic sucrose declines during infection.

**FIG 1:**
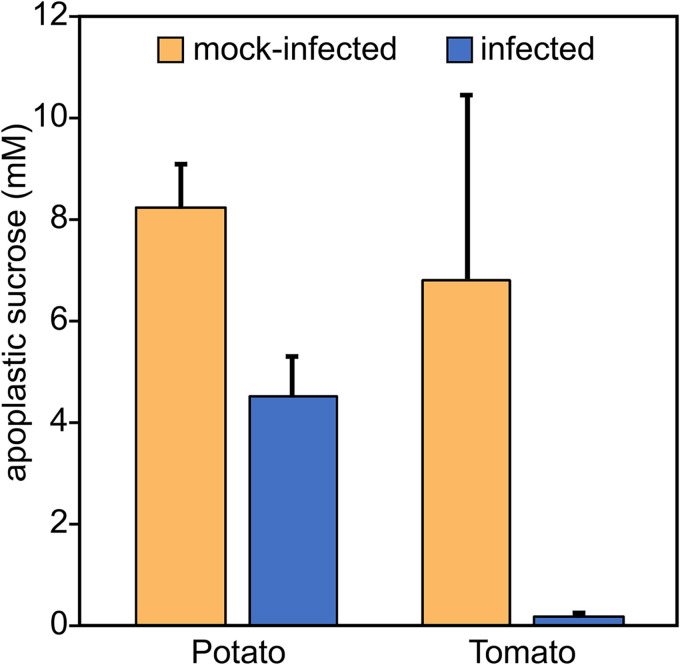
Apoplastic sucrose declines during *P. infestans* infection. Infected and mock-infected potato and tomato leaflets, after 24 h, were infiltrated with buffer. After the fluid was recovered by centrifugation, disaccharides (mostly sucrose) were quantified by liquid chromatography-tandem mass spectrometry (LC-MS/MS) using a standard curve. Values were adjusted to the true apoplastic concentration based on an indigo carmine dilution assay.

To help address which nutrients may be used by *P. infestans* during biotrophic growth, potato and tomato leaflets were infected with zoospores. After 24 h, apoplastic wash fluids were obtained by vacuum infiltration from infected and mock-infected samples. Disaccharide (mostly sucrose) levels were then quantified by liquid chromatography-mass spectrometry. Sucrose levels were found to decline sharply in infected leaflets compared to the controls (Fig. 1). This reduction may be sharper than would occur within 24 h in a natural field infection, since about 1,000 infection events occurred per leaflet in our experiment, compared to a single penetration in a normal agricultural setting.

### *P. infestans* expresses two invertases.

**FIG 2:**
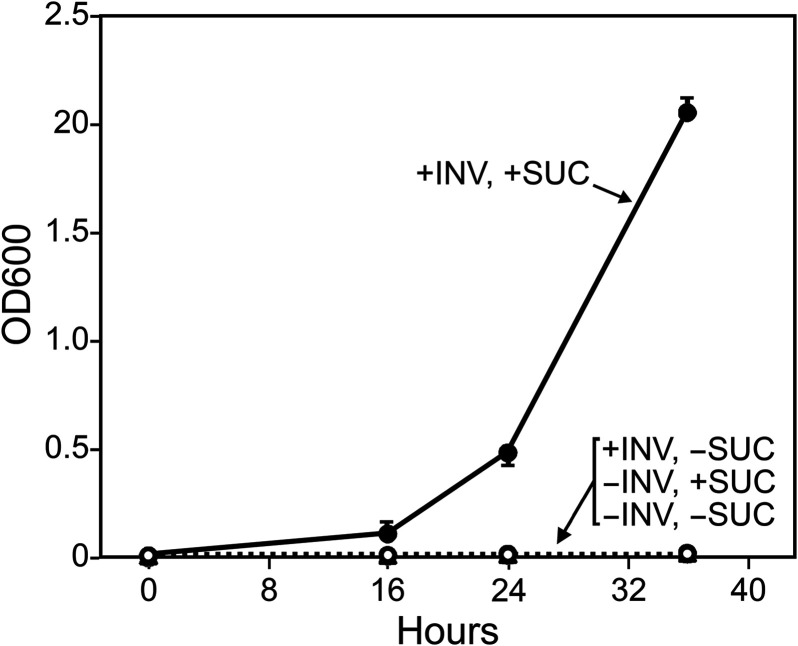
*P. infestans* invertase enables P. pastoris to use sucrose as a carbon source. P. pastoris strain KM71H and a version expressing PITG_14238 (INV) were grown on minimal medium with or without sucrose (SUC), plus methanol to induce the gene. Similar results were obtained with three independent transformants of KM71H, as well as with three independent transformants of strain X33, and when cells from methanol-induced cultures were pelleted and resuspended in methanol-free medium with or without sucrose.

Factors contributing to the drop in apoplastic sucrose during infection may include reduced photosynthesis, reduced efflux or increased uptake of photosynthate by the plant, or the action of host or pathogen invertases. To investigate the latter, a search of the *P. infestans* reference genome (strain T30-4) detected three genes encoding putative invertases. PITG_14237 and PITG_14238 were predicted to encode 68.5-kDa (617-amino-acid [aa]) and 74.4-kDa (666-aa) proteins, respectively. Transcriptome sequencing (RNA-seq) reads supported both gene models, which reside 13.3 kb from each other. Both are closely related in amino acid sequence (86% similarity), contain N-terminal signal peptides, and bear domains associated with invertases, including PFAM 00251 and PFAM 08244, which represent the N- and C-terminal domains, respectively, of glycoside hydrolase family 32. Invertase activity was confirmed by heterologous expression in Pichia pastoris, a species that lacks invertase. As shown in Fig. 2, the *P. infestans* gene allowed P. pastoris to grow using sucrose as a carbon source.

A third gene, PITG_14243, was also detected in T30-4. This was predicted to encode a 54-kDa (487-aa) protein but with truncated PFAM 00251 and PFAM 08244 domains. RNA reads from infected plant and artificial-medium samples failed to map to PITG_14243, suggesting that it is a pseudogene. An identical truncated gene was detected in an assembly of isolate 1306 of *P. infestans* (17).

### Not all oomycetes encode invertase.

**FIG 3:**
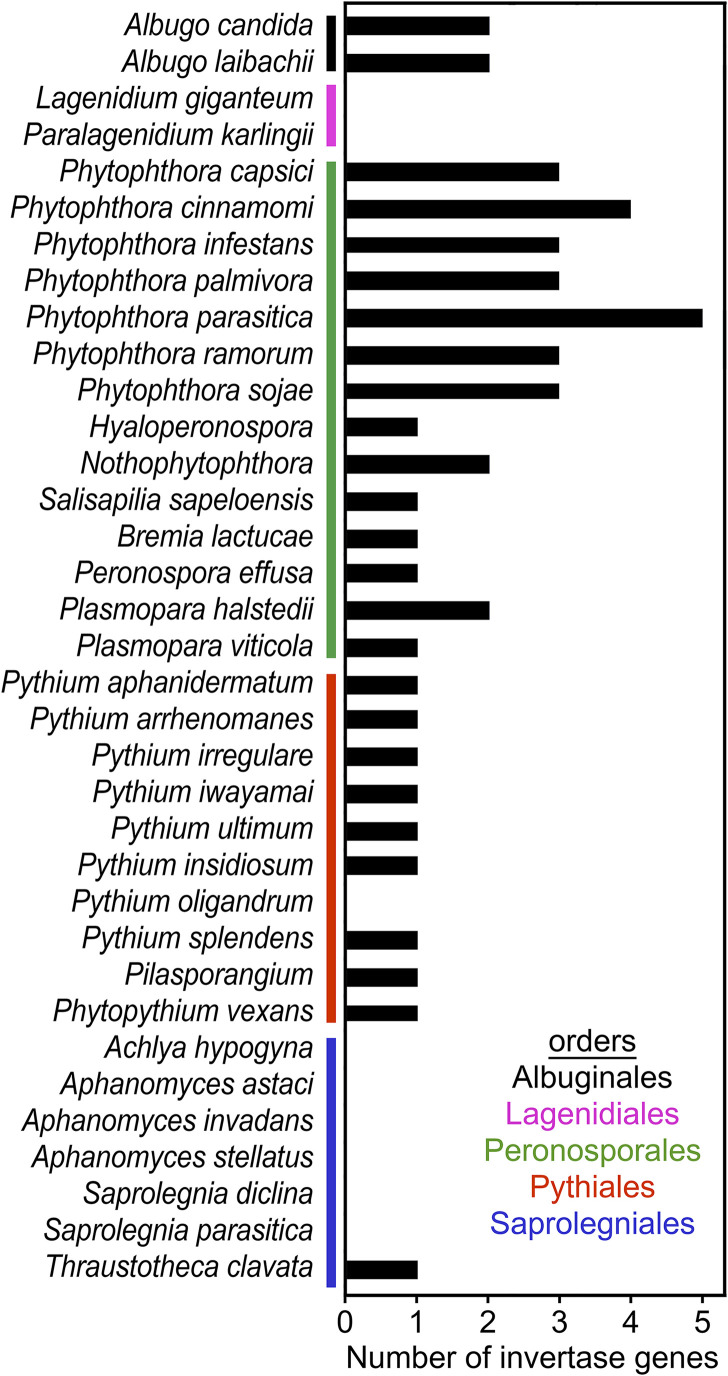
Occurrence of invertase genes in oomycetes. Genome sequences and annotated proteins were searched using representative invertases as queries in TBLASTn and BLASTP searches, using an *E* value cutoff of 10^−20^. The number shown may include some nonfunctional proteins.

The distribution of invertases was assessed using genome data from 35 species representing most oomycete orders (Fig. 3). Invertases were detected throughout the order Peronosporales. Consistent with our findings in *P. infestans*, small gene families were observed in each *Phytophthora* sp. However, no truncated genes resembling PITG_14243 were identified in the other species. A single gene was detected in Salisapilia sapeloensis, a peronosporalean that grows on plant litter. Single genes were also present in most of the obligate plant pathogens known as downy mildews (*Bremia*, *Hyaloperonospora*, *Peronospora*, and *Plasmopara*). In contrast, obligate plant pathogens of the order Albuginales (e.g., Albugo candida and Albugo laibachii) were each predicted to express two invertases.

Most representatives of the order Pythiales were found to encode a single invertase. This group mostly includes saprophytes and opportunistic pathogens of plants, or animal pests in the case of Pythium insidiosum. Phytopythium vexans, which is intermediate between the Pythiales and Peronosporales, also encoded a single invertase. Interestingly, no invertases were detected in the mycoparasite Pythium oligandrum. In this and other species lacking invertase, when possible, the searches were performed using both predicted proteins and whole-genome data from multiple strains (e.g., strains Po37 and ATCC 38472_TT for *Py. oligandrum*) (Table S1).

10.1128/mBio.01251-20.9 TABLE S1 Summed expression of enzymes in KEGG orthology groups. (A) Aggregate expression levels of each KEGG orthology group in hyphae (minimal medium), hyphae (rye-sucrose medium), sporangia, chilled sporangia, zoospores, germinated cysts, potato leaf at 2 and 6 dpi, potato leaf at 6 dpi, and tubers at 4 dpi, with the annotated enzyme and EC number for each KEGG group and the pathway classification. (B) *P. infestans* genes assigned to each KEGG group. Download Table S1, XLSX file, 0.2 MB. Copyright © 2020 Kagda et al. 2020 Kagda et al. This content is distributed under the terms of the Creative Commons Attribution 4.0 International license.

Most members of the order Saprolegniales lacked invertase. This included the three sequenced species of *Aphanomyces*, two of *Saprolegnia*, and Achlya hypogyna. These are animal or insect pathogens that also grow as saprophytes. Invertases were also absent from the order Lagenidiales, which are also insect pathogens. However, an invertase was detected in the nonpathogenic saprolegnian Thraustotheca clavata.

### *P. infestans* invertases are induced in spores and infection stages.

**FIG 4:**
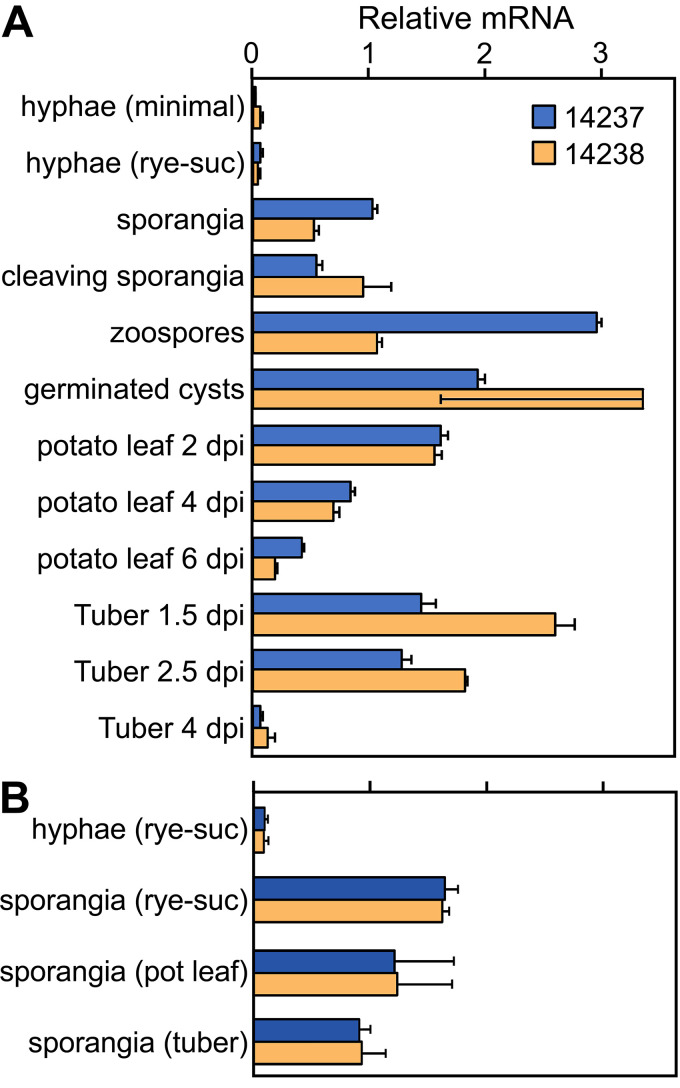
Expression of invertase genes. (A) mRNA levels of PITG_14237 and PITG_14238 were measured using RNA-seq data from young nonsporulating hyphae grown on glucose-containing minimal medium or rye-sucrose medium, sporangia from rye-sucrose (rye-suc) medium, sporangia chilled for 60 min to stimulate zoosporogenesis (cleaving sporangia), zoospores after 60 min of swimming, germinated zoospore cysts, potato leaves at 2, 4, and 6 dpi, and tubers at 1.5, 2.5, and 4 dpi. The first time point for each type of plant tissue represents biotrophic growth, and the last time point represents the necrotrophic stage. (B) mRNA levels in nonsporulating hyphae from rye-sucrose medium and sporangia isolated from rye-sucrose cultures, potato leaves, or tubers. Per-gene normalization was performed separately in each panel to a mean of 1.0.

RNA-seq revealed that PITG_14237 and PITG_14238 had similar expression patterns (Fig. 4). Their mRNA levels were very low in mycelia grown in artificial medium and collected before the onset of visible sporulation. This included nonsporulating mycelia from both minimal medium with 20 g/liter glucose and rye grain medium with 20 g/liter sucrose (Fig. 4A). The relative transcript abundance of both genes rose >10-fold in sporangia purified from rye medium cultures compared to nonsporulating mycelia and stayed elevated through germination (zoospore release) and zoospore cyst germination. In biotrophic stages of potato leaf and potato tuber infection (2 and 1.5 days postinfection [dpi], respectively), expression of both genes was >20-fold higher than in the artificial medium. Transcripts of both genes then fell to low levels as the infections entered their necrotrophic stages (6 and 4 dpi, respectively). The expression of markers for biotrophic growth, necrotrophy, and sporulation in the plant samples are shown in Fig. S1.

10.1128/mBio.01251-20.1 FIG S1 Markers of developmental stages during colonization of potato tubers and leaves. Shown are per-gene normalized expression values from RNA-seq of genes encoding RXLR protein PITG_23226, NPP1 protein PITG_16866, and centrin PITG_21764, which are markers of the biotrophic, necrotrophic, and sporulation stages, respectively (52, 61). Error bars represent standard deviations based on three biological replicates. Download FIG S1, TIF file, 0.6 MB. Copyright © 2020 Kagda et al. 2020 Kagda et al. This content is distributed under the terms of the Creative Commons Attribution 4.0 International license.

The induction of the invertases in sporangia was interesting, since we showed previously that most metabolic genes were expressed at lower levels in sporangia than mycelia (18). To ensure that the higher level of invertase mRNA in sporangia was not an artifact of growth in artificial medium, sporangia from rye-sucrose medium, potato leaf infections, and tuber infections were compared. The two invertases had similar mRNA levels regardless of their source (Fig. 4B).

### Spore, biotrophic, and necrotrophic stages form distinct clusters based on metabolic gene expression.

**FIG 5:**
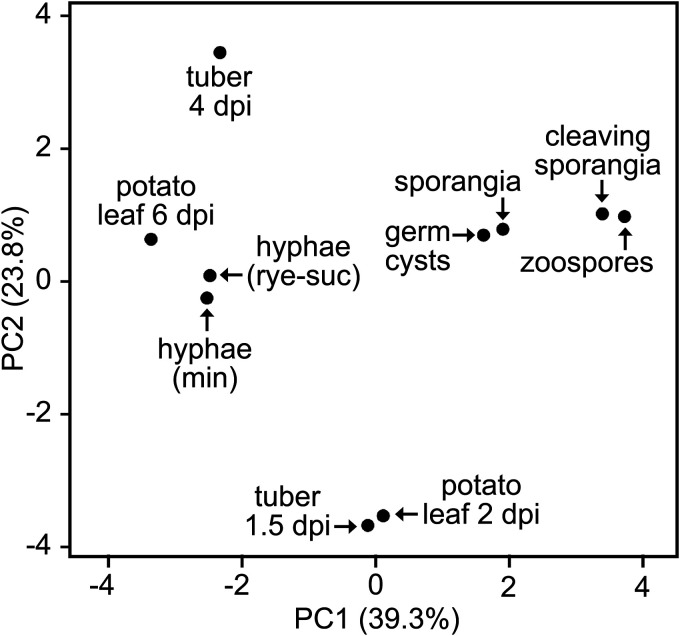
Principal-component analysis (PCA) of metabolic gene expression. The analysis was performed based on the summed FPKM values of genes in each KEGG Orthology (KO) group. Tissue samples are described in the legend to Fig. 4, and the underlying data are shown in Fig. S2.

To help understand the regulation of the invertases in the context of other metabolic enzymes, we studied the total complement of genes encoding metabolic enzymes from *P. infestans*, excluding those participating directly in protein, DNA, or cell wall modification (10). This encompassed 1,507 genes representing 586 Enzyme Commission numbers and 634 KEGG orthology groups (19). Principal-component analysis showed tight clustering of the biotrophic samples (1.5-dpi tubers and 2-dpi leaves) (Fig. 5). A second cluster included rye-sucrose medium, minimal medium, and the late stages of potato leaf and tuber infection, which indicated that metabolism during necrotrophy resembles growth on artificial medium (Fig. 5). A third cluster included sporangia, cleaving sporangia (i.e., chilled to initiate zoosporogenesis), zoospores, and germinated cysts.

10.1128/mBio.01251-20.2 FIG S2 Expression of metabolic genes classified by KEGG orthology (KO) number. RNA levels in the samples described in the legend to Fig. 3 were calculated by averaging the summed FPKM values of genes in each KO group, followed by normalization across all samples to a mean of 1. Invertase (K01193) and other enzymes mentioned in the text are highlighted by a blue arrow and diamonds, respectively. Data were subjected to hierarchical clustering using the Pearson correlation method with average linkage. The FPKM data used to make the heat map are shown in Table S1. Download FIG S2, TIF file, 2.5 MB. Copyright © 2020 Kagda et al. 2020 Kagda et al. This content is distributed under the terms of the Creative Commons Attribution 4.0 International license.

### Invertase expression is distinct from that of most other metabolic genes.

**FIG 6:**
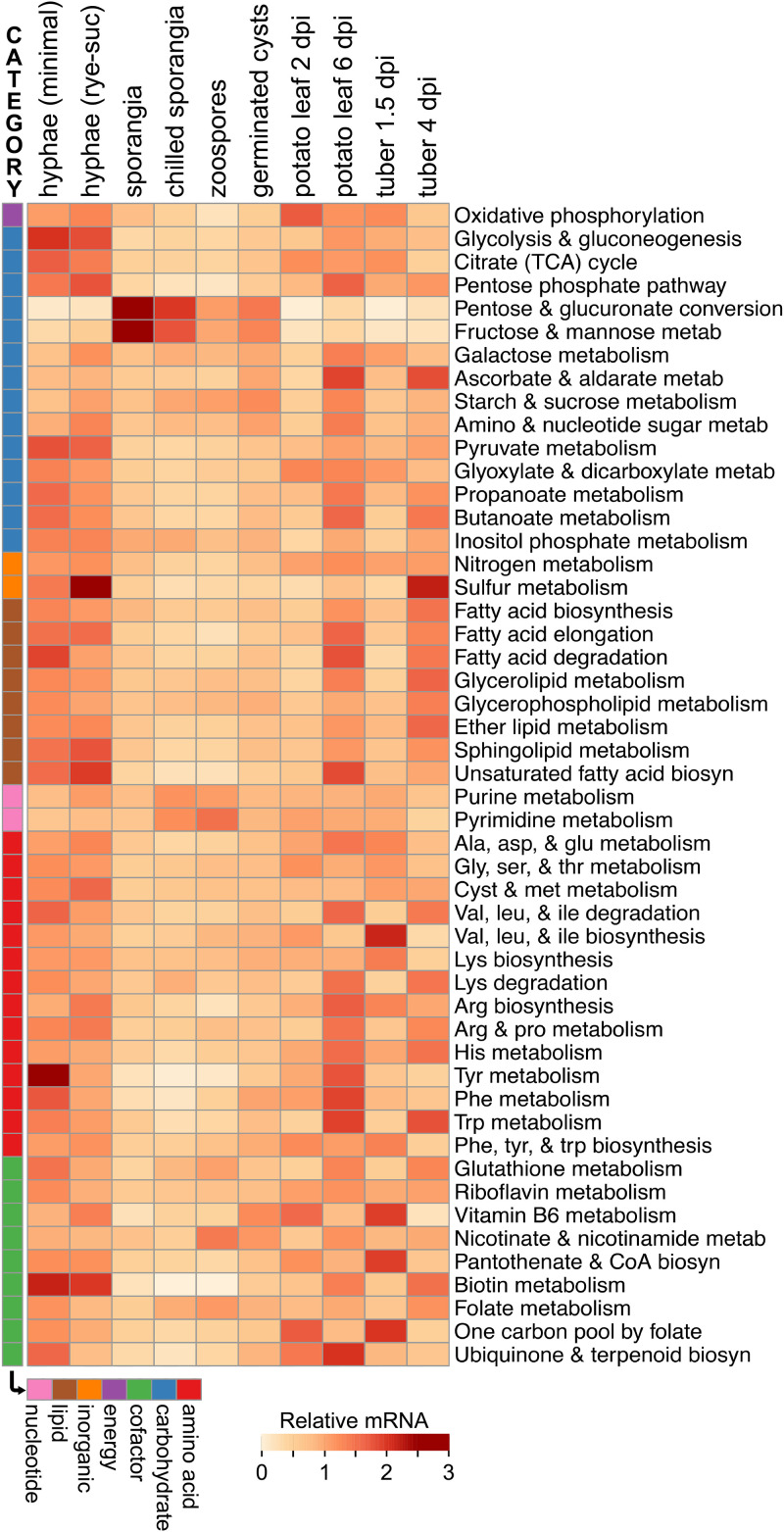
Expression of genes in metabolic pathways. Genes were grouped based on KEGG pathways, with the addition of categories for assimilating inorganic nitrogen and sulfur. The aggregate (summed) FPKM value for genes in each pathway was calculated and normalized across the tissue samples to an average of 1.0.

Analyses of KEGG pathways (ko numbers) (Fig. 6) and KEGG orthology groups (K numbers) (Fig. S2) further demonstrated that the expression profile of the invertases was unusual. We first examined the summed FPKM (fragments per kilobase per million) values of genes in the 50 major metabolic pathways defined by KEGG. Invertases (K01193) are included, along with multiple other enzymes, in the KEGG pathways for galactose metabolism (ko00052) and starch and sucrose metabolism (ko00500). Transcript levels of most pathways were lower in the spore stages than during growth in plants or medium, which is opposite to that observed for the invertases (Fig. 6). Four pathways were upregulated in spores. The fructose/mannose metabolism and pentose/glucuronate conversion pathways (KEGG pathways ko00040 and ko00051) were higher in spores, primarily due to spore-specific sugar alcohol dehydrogenases in KEGG orthology group K00008. Pyrimidine and purine metabolism (ko00230 and ko00240) were higher in spores due to upregulated biosynthetic enzymes such as IMP dehydrogenase (K00088) and others. Also upregulated in spores were ribonucleoside-diphosphate reductase subunits (K10807 and K10808), which may be related to preparing for DNA synthesis after germination, and cyclic nucleotide phosphodiesterases (K01120), which may participate in signal transduction during zoosporogenesis (20). Unlike invertases, none of these were expressed more during biotrophic (early) than necrotrophic (late) stages of infection.

About one-third of KEGG pathways were transcribed more during early than late infection. Examples include the oxidative phosphorylation (ko00750) and vitamin biosynthesis pathways (e.g., ko00190 and ko00770). However, unlike invertases, these were downregulated in spores.

Similar conclusions about the atypical nature of invertase expression were drawn by inspecting the 634 KEGG orthology groups encoding metabolic enzymes (Fig. S2; Table S1). When the average expression in spore stages was compared to the average in the mycelia from artificial medium, only 16 and 25 groups were higher in spores based on 10-fold and 4-fold cutoffs, respectively. Besides those mentioned above, these included tyrosinase (K00505), sugar epimerase (K08679), ceramidase (K04711), carnitine O-palmitoyltransferase (K08765), and two cofactor biosynthesis enzymes, nicotinamidase (K08281) and protoporphyrinogen oxidase (K00231). However, few were expressed more during early than late infection. Instead, most spore-induced genes had higher mRNA levels during late infection, probably since this is when sporulation initiates. Examples include ceramidase and carnitine O-palmitoyltransferase, which might mobilize carbon from the lipid reserves of spores.

About 69% of the 634 KEGG orthology groups showed higher expression during late versus early infection in leaves and tubers, with 16% showing the opposite pattern (Fig. S2). Nearly all of the latter, however, differed from invertases by not being upregulated in spores compared to medium. Only a few KEGG orthology groups (<1%) had patterns of expression that roughly matched the invertases. These included mostly cytoplasmic enzymes, including a threonine catabolic enzyme (K15789) and the ketogenic enzyme hydroxymethylglutaryl-CoA lyase (K01640).

### Invertase expression resembles that of only a few other secreted proteins.

We predicted that approximately 40 types of metabolic enzymes (encoded by 111 genes with an FPKM value of >1) were secreted. Interestingly, mRNA levels of 28% of these genes were >10-fold higher in one or more spore stages (sporangia to germinated cysts) than mycelia from artificial medium. Also, 22% were >10-fold higher in one or more plant tissues than mycelia from medium (Fig. S3). Fifteen genes were expressed at >10-fold-higher levels both in a spore stage and during plant infection compared to artificial medium, including the invertases. These included three carbonic anhydrases, three acid phosphatases, an aldose epimerase, two phospholipases, one tyrosinase, a β-glucosidase, and a catalase. Hierarchical clustering indicated that the catalase and carbonic anhydrases were closest in expression pattern to the invertases.

10.1128/mBio.01251-20.3 FIG S3 Expression of secreted metabolic enzymes. Indicated are per-gene-normalized expression values based on the samples described in the legend to Fig. 3. Each gene is annotated with its function, Enzyme Commission (EC) number, and gene name (PITG_XXXXX). The two invertases are highlighted with arrows. Data were subjected to hierarchical clustering using the Pearson correlation method with complete linkage. Download FIG S3, TIF file, 1.5 MB. Copyright © 2020 Kagda et al. 2020 Kagda et al. This content is distributed under the terms of the Creative Commons Attribution 4.0 International license.

We also checked whether genes encoding nonmetabolic secreted proteins traditionally associated with plant-pathogen interactions had mRNA profiles similar to those of invertase genes. Only 4 of the 312 expressed genes for RXLR effectors resembled invertases in being upregulated in spores and in early compared to late infection in both leaves and tubers (e.g., PITG_02843) (Fig. S4). Interestingly, many of the RXLRs were differentially expressed between the two types of host tissues. In contrast, no cell wall-degrading enzymes (Fig. S4) or proteases (Fig. S5) had expression patterns that resembled those of the invertases. It was nevertheless interesting to observe that many of these were transcribed at much higher levels in tubers than in leaves.

10.1128/mBio.01251-20.4 FIG S4 Expression of secreted RXLR and protease effectors. Data are per-gene-normalized expression values based on the samples described in the legend to Fig. 3. Invertase is marked by the arrow. Data were subjected to hierarchical clustering using the Pearson correlation method with average linkage. Download FIG S4, TIF file, 1.8 MB. Copyright © 2020 Kagda et al. 2020 Kagda et al. This content is distributed under the terms of the Creative Commons Attribution 4.0 International license.

10.1128/mBio.01251-20.5 FIG S5 Expression of cell wall-degrading (CWDE) enzymes. Data are per-gene-normalized expression values based on the samples in Fig. 3. Appended to the name of each gene is its enzymatic function, as follows: CUT (cutinase); PE (pectin esterase, carbohydrate esterase family CE8); pectate lyase families PL1, PL2, and PL3; GH28 (polygalacturonase); GH53 (endo-β-1,4-galactanase); GH54 (β-xylosidase); GH78 (α-l-rhamnosidase); and GH105 (rhamnogalacturonyl hydrolase). Invertase expression is marked by the arrow. Data were subjected to hierarchical clustering using the Pearson correlation method with average linkage. Download FIG S5, TIF file, 1.9 MB. Copyright © 2020 Kagda et al. 2020 Kagda et al. This content is distributed under the terms of the Creative Commons Attribution 4.0 International license.

### Some sugar transporters are induced in spores and plants.

Since invertases are expected to liberate monosaccharides for use by *P. infestans*, we also examined sugar transporters (Fig. S6). Of 44 genes expressed with FPKM values of >1, none exhibited patterns similar to those of the invertases. A majority were expressed at higher levels during late infection than early infection. One gene encoding a SWEET/MtN3 transporter (PITG_04173) was expressed preferentially at early time points in both leaves and tubers, like the invertases. However, PITG_04173 was also expressed at similar levels in artificial medium and plants.

10.1128/mBio.01251-20.6 FIG S6 Expression of predicted sugar transporters. Data are per-gene-normalized expression values based on the samples described in Fig. 3. Genes belong to the MFS or MTN3 (SWEET) families, as noted. Invertase is marked by the arrow. Data were subjected to hierarchical clustering using the Pearson correlation method with average linkage. Download FIG S6, TIF file, 1.6 MB. Copyright © 2020 Kagda et al. 2020 Kagda et al. This content is distributed under the terms of the Creative Commons Attribution 4.0 International license.

### *P. infestans* invertases are not induced by sucrose.

**FIG 7:**
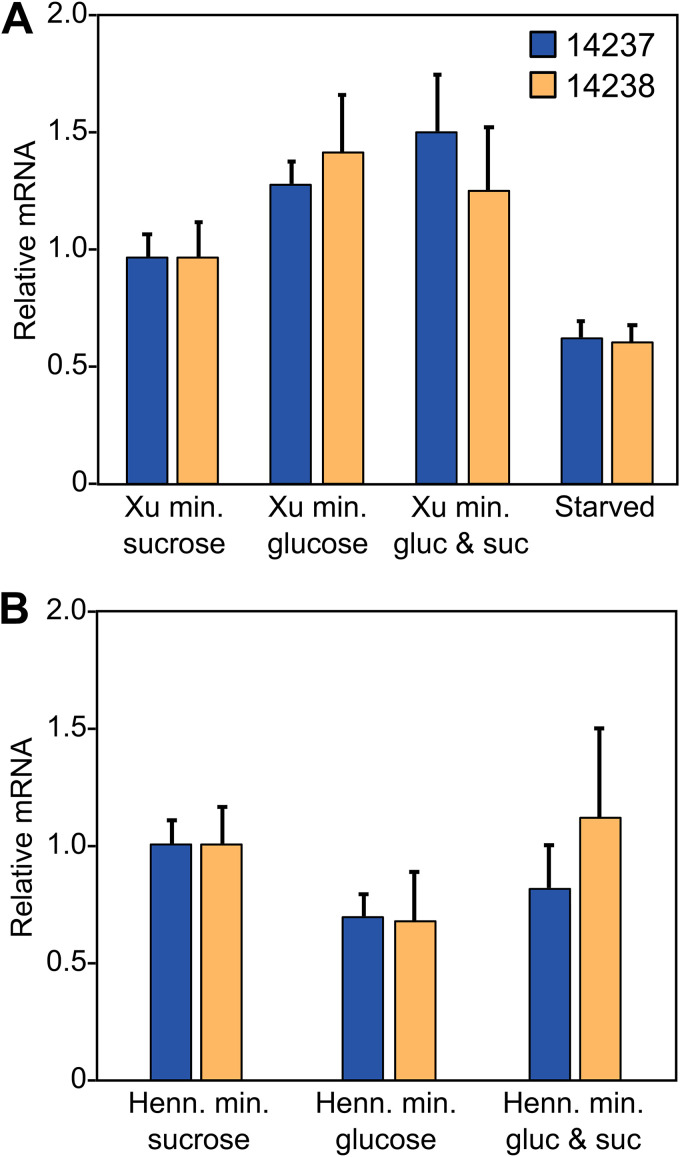
Effect of sugars on expression of *P. infestans* invertase genes. (A) qRT-PCR analysis of the invertases in young (nonsporulating) hyphae from modified Xu’s minimal medium containing 20 g/liter sucrose, glucose, or both sugars. Also shown are values for mycelia grown in sucrose but then starved for 6 h in medium lacking a carbon source. Expression values were calculated by the ΔΔ*C_T_* method, using two constitutive genes as a control, and normalized to the samples containing only sucrose. (B) Same as panel A except that modified Henninger’s medium was used.

Fungal invertases are typically upregulated by sucrose (14, 15). Although such data come mostly from studies of saprophytic fungi, it was nevertheless surprising that the *P. infestans* invertases were transcribed at similarly low levels in both the glucose-based minimal medium and the sucrose-based rye grain medium (Fig. 4). To better ascertain whether sucrose induces the invertases, their expression was measured by RT-qPCR in two distinct types of minimal medium (21, 22), each modified to include glucose, sucrose, or both (Fig. 7). PITG_14237 and PITG_14238 exhibited similar mRNA levels regardless of the sugar, indicating that sucrose did not induce either invertase. We also considered whether nutrient limitation might upregulate the invertases, since sporangia are metabolically active but reliant on carbon reserves. However, placing mycelia for 6 h in medium lacking a carbon source did not induce either gene.

We also attempted to test the effect of fructose, which is the other end product of the invertase reaction. This was not feasible, however. With Xu’s minimal medium (21), no growth was observed when fructose or a combination of fructose and sucrose was used. With Henninger’s medium (22), some growth occurred with fructose but very slow growth occurred when both sugars were present.

### Invertase accumulates in haustoria.

**FIG 8:**
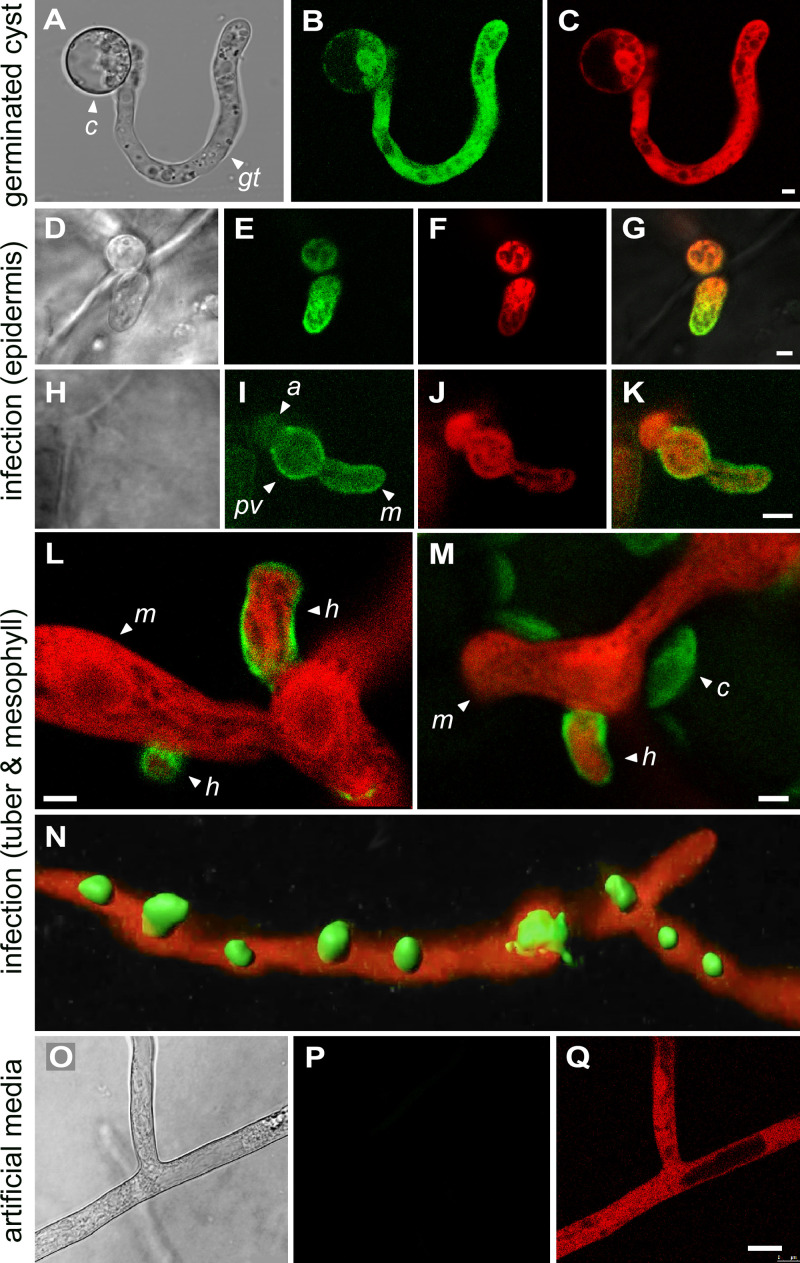
Localization of invertase expressed from native promoter. (A to C) Germinating zoospore cyst from strain expressing invertase fused with GFP and a separate gene encoding cytoplasmic tdTomato. Panels show bright-field, green, and red channels, from left to right. The cyst (c) and germ tube (gt) are marked. (D to G) Strain coexpressing invertase-GFP and cytoplasmic tdTomato in tomato leaf epidermal cell. The appressorium (a) and a young intracellular mycelium (m) are marked. Panels show bright-field, green, red, and merged images, from left to right. (H to K) Similar to panels D to G but at a later stage of growth. An appressorium (a), intracellular primary vesicle (pv), and mycelium (m) are marked. (L) Strain expressing invertase-GFP and cytoplasmic tdTomato in the medulla of an infected potato tuber. Emerging from the mycelium and penetrating host cells are several haustoria (h). (M) Same as panel L but showing mesophyll of a tomato leaf. The green channel detects both a GFP signal surrounding the haustorium (h) and autofluorescing chloroplasts (c). (N) Similar tissue as in panels L and M but showing a z-stack reconstruction of haustoria formed in the plant using Imaris software. (O to Q) Same strain as panel N but illustrating growth in rye-sucrose medium, showing an absence of invertase-GFP expression. Panels show bright-field, green, and red channels, from left to right. Each labeling pattern was observed in a minimum of three transformants. The images were obtained with PITG_14238, but similar results were obtained with PITG_14237. Bars, 2.5 μm.

To study the location of the protein, we generated transformants of *P. infestans* expressing the invertases fused to green fluorescent protein (GFP), driven by the native promoters. Even though mRNA from both invertase genes was detected in sporangia, no invertase-GFP was detected by confocal microscopy in ungerminated sporangia ranging in age from 1 to 5 days. It is possible that not all mRNA in that dormant stage is efficiently translated. However, a GFP signal was detected in germinated cysts (Fig. 8A to C). This transformant, and those in the subsequent panels of Fig. 8, coexpress invertase-GFP from the native promoter along with cytoplasmic tdTomato driven by the constitutive *ham34* promoter. Invertase-GFP was distributed throughout the cytoplasm of germinated cysts, even though our constructs included the N-terminal signal peptide. This might be explained by the fact that secreted proteins would be below detection levels due to dilution in the medium.

During the initial stages of penetration of an epidermal cell of a tomato leaf, invertase-GFP continued to show a cytoplasmic signal (Fig. 8D to G). However, instead of being uniformly distributed, it accumulated preferentially at the hyphal tip. At a later stage of development in epidermal cells, soon after formation of the so-called primary vesicle, invertase-GFP accumulated in the pathogen cell wall in addition to the cytoplasm (Fig. 8H to K). During subsequent growth in the medulla (flesh) of potato tubers or tomato leaf mesophyll, invertase-GFP resided mostly in the haustorial cell wall (Fig. 8L and M).

While the invertase-GFP signal was not difficult to observe in cyst germ tubes or haustoria, the same transformants exhibited little signal in hyphae in artificial medium (Fig. 8O to Q). This is consistent with the expression patterns in Fig. 6. Patches of invertase-GFP were seen occasionally in older cultures, which we assume represent zones in which sporulation was occurring.

In contrast, when invertase-GFP was expressed from the constitutive *ham34* promoter (23) in artificial medium, the protein was detected in hyphal walls (Fig. S7A and B). It also concentrated in sporangial papilla (Fig. S7C and D). Since we showed previously that fluorescent proteins lacking signal peptides are cytoplasmic in sporangia (24), we hypothesized that all secreted proteins might accumulate in papilla. As shown in Fig. S7, this appeared to be true in transformants in which the *ham34* promoter drove the expression of tdTomato fused to 33 amino acids from the N-terminal region of the secreted *P. infestans* INF1 protein (25), which includes the 20-amino-acid signal peptide predicted by TargetP. Although the papillar location of secreted proteins does not appear to be relevant to the invertase story, papilla have been suggested to contain enzymes that facilitate germination (26).

10.1128/mBio.01251-20.7 FIG S7 Expression of invertase in rye-sucrose medium using *ham34* promoter. (A) Hypha of *P. infestans* transformant expressing invertase-GFP (showing expression in the cell wall [w]) and mCherry-tagged histone H2b as a marker of nuclei (red channel [n]). (B) Sporangium from *P. infestans* strain expressing invertase-GFP, showing strong fluorescence at papilla (p). (C) Sporangium of strain expressing invertase-GFP and histone H2b-mCherry. (D) Strain expressing tdTomato fused to the *Inf1* signal peptide. Download FIG S7, TIF file, 2.7 MB. Copyright © 2020 Kagda et al. 2020 Kagda et al. This content is distributed under the terms of the Creative Commons Attribution 4.0 International license.

### Haustoria function as secretion organelles.

**FIG 9:**
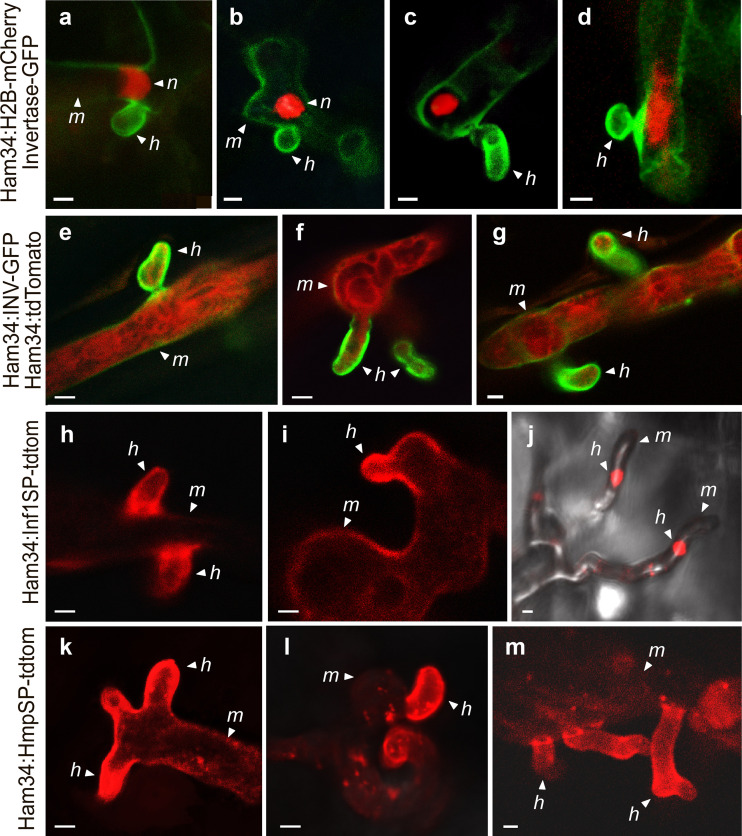
Investigating basis of haustoria targeting. (a to d) Transformants coexpressing invertase-GFP with H2B-mCherry during tuber infection. Nuclei (n), haustoria (h), and intercellular mycelia (m) are marked. (e to g) Transformants coexpressing cytoplasmic tdTomato and invertase-GFP in tubers, but unlike in Fig. 8, data were obtained using the *ham34* promoter. (h to j) Transformants expressing a fusion of the N-terminal region of INF1 containing its signal peptide with tdTomato, driven by the *ham34* promoter, in tuber infection. Note that in panel j, the haustoria extend upward from the hyphae. (k to m) Transformants expressing a fusion of the N-terminal region of HMP1 containing its signal peptide and tdTomato in tubers. Bars, 2.5 μm.

It was interesting that most invertase during infection resided in the haustorial wall, based on transformants expressing invertase-GFP from their native promoters (Fig. 8). Since we observed that nuclei typically reside adjacent to haustoria (Fig. 9a to d), we considered the possibility that invertase promoters are specifically active in those nuclei. However, preferential deposition of the protein in haustoria also occurred when invertase-GFP expression was driven by *ham34* (Fig. 9e to g).

An alternative theory to explain the haustorial accumulation of invertase is that all secreted proteins are delivered to that structure. This would be consistent with a recent study that showed that diverse extracellular infection-associated proteins also accumulated near haustoria (27). We tested this further by using the *ham34* promoter to express tdTomato fused to 33 amino acids from the N terminus of the *P. infestans* INF1 protein, which is downregulated during biotrophic growth and thus not associated with haustoria (25), or 42 N-terminal amino acids from HMP1, which is a known haustorial protein (11). Within these regions, both INF1 and HMP1 contain canonical signal peptides based on TargetP (Fig. S8) (28). Both tdTomato fusions accumulated preferentially in haustoria (Fig. 9h to m).

10.1128/mBio.01251-20.8 FIG S8 N-terminal regions of proteins mentioned in this paper. The first 64 amino acids of the INF1 (PITG_12251), HMP1 (PITG_00375), and invertase (PITG_14238) proteins are shown. Grey shading indicates the signal peptides predicted by TargetP 2.0 (28). The regions used in tdTomato fusions involving INF1 and HMP1 are underlined. PITG_21410, PITG_22926, PITG_01029, and PITG_14371 are the secreted INF4, pectinesterase, and RXLR proteins, which were shown in other studies to concentrate near haustoria (27). Sequences C-terminal of the signal peptide are dissimilar in chemical features such as hydropathy, ranging from moderately hydrophilic to hydrophobic, and lack conserved motifs based on alignments and motif discovery programs. Download FIG S8, PDF file, 0.05 MB. Copyright © 2020 Kagda et al. 2020 Kagda et al. This content is distributed under the terms of the Creative Commons Attribution 4.0 International license.

## DISCUSSION

Unlike characterized fungal invertases, those of *P. infestans* are not sucrose inducible but instead are expressed primarily during preinfection stages (spores) and biotrophic growth (Fig. 4). That the substrate-induction paradigm does not extend to *P. infestans* might seem surprising, since oomycetes acquired invertases by lateral transfer from fungi (8). Expression neofunctionalization is understandable if lifestyle differences are considered, however. Most *Phytophthora* spp. depend on a living host for survival, as they survive poorly as saprophytes except for some clade 6 species (*P. infestans* is clade 1c) (29). In contrast, most fungal pathogens alternate between saprophytic and pathogenic growth in nature. Since the normal environment of *P. infestans* is a sucrose-bearing plant, a mechanism for regulation based on substrate induction would be superfluous. It is more logical to trigger invertase transcription in spores to allow sucrose to be used immediately upon invasion of the host. Similar reasoning justifies the expression of a subset of plant defense-suppressing RXLR proteins and other effectors in spores (Fig. S4 and S5).

In contrast to invertase, most metabolic genes and pathways were downregulated in spores compared to artificial medium or infected plants (Fig. 6 and Fig. S2). By mining public proteomic data (30) for 35 randomly selected enzymes in such pathways (glycolysis, oxidative phosphorylation, pentose phosphate pathway, amino acid metabolism), we observed that their protein levels decline an average of 34% between mycelia and germinating cysts; this residual amount is apparently enough to allow successful pathogenesis. That study focused on cytoplasmic proteins and thus did not yield data on invertase.

The novelty of the pattern of invertase expression in *P. infestans* versus characterized nonoomycetes is supported by a survey of the literature and our mining of public RNA-seq data. For example, while little expression of the *P. infestans* invertases occurred in artificial medium compared to plant infection, no significant difference between medium and *in planta* growth was observed in the rust fungus *Uromyces fabae* and the bacterium Erwinia amylovora (31, 32). Moreover, while *P. infestans* invertases were expressed primarily during early infection, those of the biotroph Ustilago maydis and hemibiotroph Fusarium graminearum were expressed at higher levels during late infection (33, 34). Whether the *U. maydis* and F. graminearum invertases are developmentally regulated or induced in response to a rise in sucrose during late infection is unknown. This would be interesting to explore, since most research on fungal invertases has involved saprophytes, not host-adapted pathogens.

In *P. infestans*, extracellular carbonic anhydrases were among the few enzymes that exhibited transcriptional patterns resembling those of invertases, i.e., upregulation in spores and during biotrophy (Fig. S3). By converting carbon dioxide and water to bicarbonate and protons, carbonic anhydrases are candidate effectors that may acidify the haustorial interface to benefit the pathogen. It is interesting that the invertase of Phytophthora palmivora was shown to function optimally at a slightly acidic pH (35). Our analysis may underestimate the number of secreted enzyme activities that, like carbonic anhydrases, are coexpressed with invertases. For example, although distinct aldose epimerases were spore or biotrophy induced, their aggregate activity profile may match that of invertase. These secreted epimerases may work in concert with invertase by speeding the anomeric conversion of α- to β-d-glucose. *P. infestans* glucokinases are believed to be of bacterial origin, in which the enzymes more efficiently bind the β form (36).

The idea that oomycete haustoria participate in nutrient uptake is supported by our localization of invertases to that structure (Fig. 8). We hypothesize that invertase targeting does not require any motif other than its signal peptide; haustoria are simply the default destination for *in planta*-secreted proteins. Several data support this proposition. First, a prior study showed that three diverse extracellular infection-related proteins were all secreted from haustoria (27). Second, we found that tdTomato fused to the N-terminal regions of INF1 and HMP1 accumulated in haustoria (Fig. 9). Since our constructs included a few amino acids C-terminal to the signal peptide and since the N-terminal regions of only a few proteins were tested, additional studies could test if the requisite sequences are entirely within the signal peptide, if a second motif related to haustorial delivery resides within the signal peptide, and if N-terminal peptides from additional proteins provide the same result. We did examine the N-terminal regions of the haustoria-delivered proteins studied here and in a previous study (27) (Fig. S8) but found no shared characteristics (sequence, hydrophobicity, or secondary structure) other than those diagnostic of a signal peptide.

The concentration of *P. infestans* invertase in the haustorial wall is consistent with a prior study of Phytophthora sojae in medium that showed that its invertase is a glycoprotein, with >50% of the activity bound to the cell wall (37). We also observed invertase-GFP in the cell walls of *P. infestans* hyphae in medium when the transgene was expressed from the constitutive *ham34* promoter (Fig. S7). Cell walls are known to trap many secreted proteins, especially glycoproteins, which include most fungal and plant invertases (38–40). PITG_14237 and PITG_14238 contain predicted N-glycosylation sites, particularly C-terminal to their GH32 (PFAM 00251) domains. However, using the native promoter, we observed that the invertase-GFP signal in germinating cysts was cytoplasmic. This might be explained if protein secretion by young germinated cysts is slow, if the wall signal becomes obvious only after a more extended time of secretion, or if protein glycosylation or cell wall structure in cysts differs from that of more mature hyphae. The latter is consistent with our observation that β-glucanases digest the walls of cyst germ tubes faster than those of more mature hyphae (41).

Although they are known to serve as a location for enzyme secretion, the full scope of involvement of oomycete haustoria in nutrition requires further investigation, since the site of glucose uptake is unknown. Unlike the case in fungi (7), no sugar transporter of *P. infestans* was transcriptionally upregulated in the haustoria-forming phases of potato leaf and tuber colonization (Fig. S5). Oomycete haustoria also lack a neckband, which encircles fungal haustoria and may help establish electrochemical gradients that aid nutrient transport (42). Instead, the extrahaustorial matrix of *P. infestans* is continuous with the rest of the apoplast (43). Consequently, hexoses generated by invertase may diffuse from the haustorial interface to transporters at other locations. Also, some invertase may escape the haustorial wall and become dispersed to other regions of the apoplast.

Regardless of their precise location, the combination of hexose transporters and invertases would improve the sink strength of *P. infestans*, providing carbohydrate to enhance pathogen growth. The relative contributions of the two types of proteins cannot be measured at present. Although *ex planta* studies suggest that glucose is a preferred carbon source (44), other apoplastic compounds may also supply carbon. If *P. infestans* has sugar transporters that can outcompete host transporters, its invertase may be dispensable, as suggested for Ustilago maydis (45). We have not yet succeeded at generating invertase knockdown strains of *P. infestans* using homology-based silencing methods that we have used successfully for other genes. Thus, evidence for a role of the *P. infestans* invertases in pathogenesis remains indirect. Nevertheless, the taxonomic distribution of invertases in oomycetes argues in favor of their contribution to plant pathogenicity: most oomycetes that infect animals also lack invertase (Fig. 3). Also, the *P. infestans* invertases likely contribute to the decline in apoplastic sucrose during leaf infection (Fig. 1), although enhanced activity of plant cell wall invertases or sugar transporters may also be responsible (46). It is well-known that some plants intensify sugar uptake during infection, which may reduce nutrients available to pathogens (47). In *Arabidopsis*, for example, sugar transporter STP13 was shown to contribute to resistance against both fungal and bacterial pathogens (48, 49). The movement of sugars into plant cells may also explain why we previously observed only small decreases in total, as opposed to apoplastic, leaf sucrose early in infection (50).

## MATERIALS AND METHODS

### *P. infestans* growth and development.

Strain 1306 was maintained in the dark in rye-sucrose agar (51) at 18°C. For RNA analysis, cultures were grown in rye-sucrose medium, minimal medium based on the recipe of Xu (21) using 0.11 M glucose and/or sucrose as the carbon source, or Henninger’s minimal medium (22) using a total of 0.055 M concentrations of the sugar(s). Nonsporulating mycelia were from 3-day cultures. Spore stages were obtained from plants as described below or rye medium (18). For the latter, sporangia were scraped from 10-day cultures using water and a glass rod and frozen pending RNA extraction or chilled at 10°C for 60 min to initiate zoosporogenesis (sporangial cleavage). Zoospores were obtained by incubating sporangia for an additional 90 min. Germinating cysts were made by adding 0.25 mM CaCl_2_ to zoospores, vortexing for 1 min, and incubating the resulting cysts for 6 h in water at 18°C. Starved hyphae were obtained by washing mycelia grown in rye medium twice in modified Petri’s solution (52) and then incubating the hyphae for a further 6 h in Petri’s solution.

### Plant infection.

Tubers (cv. Russet Burbank) were washed in water, soaked in 10% (vol/vol) household bleach for 15 min, rinsed in water, and cut into 2-mm slices. After a second rinse, the slices were blotted dry and placed on a rack 8 mm above moist towels in a sealed box. Inoculations were performed by spreading about 0.2 ml of a 5 × 10^5^/ml zoospore suspension per slice. These were kept at 18°C in the dark and harvested after 1.5, 2.5, and 4 days.

Leaf infections used potato cv. Russet Burbank or tomato cv. New Yorker grown using 14-h days (400 μmol m^−2^ s^−1^) at 25°C and 10-h nights at 18°C. Prior to infection, plants were equilibrated in a chamber using 12-h days (200 μmol m^−2^ s^−1^) and 12-h nights at 18°C. For RNA-seq, whole plants were sprayed with 10^5^/ml zoospores to the point at which runoff occurred, placed in a plastic bag, and incubated in the 18°C chamber. For analyses of apoplastic sucrose, detached leaflets were inoculated with approximately 0.2 ml of water or zoospores at 2 × 10^4^/ml. These were placed in the 18°C chamber in humidified boxes.

Sporangia for RNA-seq were washed from tubers or leaves and collected by centrifugation. Apoplastic wash fluids from leaves were prepared using modifications of existing methods (53, 54). This involved vacuum infiltration, wrapping in plastic film, and centrifugation at 1,750 × *g* at 4°C above a plastic support. Tissue integrity was assessed using propidium iodide (54) and by measuring β-glucose-6-phosphate. Metabolite concentrations were determined by the UCR Metabolomics Core Facility using external and internal standards, with a Waters TQ-XS triple quadrupole mass spectrometer coupled to a two-dimensional ultraperformance liquid chromatograph (UPLC).

### *P. infestans* transformants.

Coding sequences plus 500 nucleotides (nt) of promoters were amplified using Platinum High Fidelity polymerase (Invitrogen), cloned into pGEMT-Easy (Promega), and subcloned in place of the *ham34* promoter in pGFPH (hygromycin resistance) (24) to make invertase-GFP fusions. Other constructs had the coding sequences downstream of the *ham34* promoter. In some experiments, these plasmids were cotransformed into *P. infestans* with pTdTomatoN or pH2B-TdTomato (G418 resistance). Other vectors were constructed by making translational fusions of the N-terminal regions of INF1 or HMP1 with TdTomato in pTdTomatoN (Fig. S8). Vectors were transformed into *P. infestans* by the protoplast method (24) using 12 μg/ml G418 and 50 μg/ml of hygromycin.

Confocal microscopy was performed using tissue from medium, tomato leaves (cv. New Yorker), or tuber slices (cv. Yukon Gold). Just before examination, tuber slices were cut into 5-mm cubes, while 5- by 5-mm squares were cut from leaves. These were inverted (infection side down) on 35-mm glass-bottom culture dishes (MatTak) under a cover glass. Other samples were mounted on a slide. Microscopy was performed using a Leica SP5 laser scanning inverted confocal microscope with a 40× water objective, using fluorescein isothiocyanate (FITC; 488 nm; for GFP) and tetramethyl rhodamine isocyanate (TRITC; 543 nm; for tdTomato) channels at 15% and 33% intensity, respectively. Two-color images were obtained using sequential scanning to prevent bleed-through.

### P. pastoris experiments.

Strains KM71H and X33 were used to express *P. infestans* invertase using the EasySelect *Pichia* expression kit (Thermo). This involved cloning the mature peptide region of PITG_14238 behind the yeast signal peptide in pPICZα. Growth assays were performed at 30°C using shaking cultures in minimal medium (0.1 M potassium phosphate [pH 6.0], 13.4 g/liter yeast nitrogen base without amino acids, 40 pg/liter biotin, 5 ml/liter methanol, 40 mg/ml arginine) supplemented as appropriate with 10 g/liter sucrose.

### RNA analysis.

For RNA-seq, experiments with plant samples used three biological replicates, while *ex planta* studies of development had two replicates. RNA was isolated using the Sigma Plant Total RNA kit. Single-end reads (75 nt) were aligned and mapped using HiSat2, and expression calls were made with edgeR, using systemPipeR (55). Low-expression-level genes were eliminated using a FPKM threshold of 1.0. Hierarchical clustering, heat map generation, and PCA used ClustVis (56). A minimum of 25 million reads were obtained per replicate of pure (nonplant) *P. infestans* samples, while 250, 160, and 80 million reads were obtained for early, middle, and late leaf samples and 1,000, 820, and 920 million reads were obtained for tuber samples.

RT-qPCR was performed as described elsewhere (52) using primers specific for PITG_14237 (5′-GTGCCAGCCAGACGAAGTC and 5′-GTGCTTAGGCTCCGTCCAGA) and PITG_14238 (5′-ACGGCATCAGGTAAGACCAC and 5′-CGACCTTCCCAGTCTTCTTG). cDNA made using the Maxima First-Strand kit (Thermo) was analyzed using a CFX Connect system (Bio-Rad) using the Dynamo HS SYBR green kit (Thermo). Assays employed two biological replicates, each with three technical replicates. Reverse transcriptase-lacking controls and melt curves confirmed the fidelity of amplification. Gene expression was determined by the ΔΔ*C_T_* method using experimentally determined primer efficiencies with two constitutive genes as a reference. These were PITG_11766, which encodes ribosomal protein S3A, and PITG_09862, which encodes a Kelch repeat protein (57).

### Sequence analysis.

Functions were assigned to metabolic proteins as described elsewhere (10). In brief, protein sequences were used as inputs for the KEGG-based KAAS and BlastKoala annotation servers. Annotations were checked for consistency with results from the Conserved Domain Database (CDD) and BLASTP searches of GenBank. Enzymes were placed into pathways based on the KEGG classification. Secreted proteins were identified using SignalP 4.1 and TargetP (28); genes were excluded if they contained a KDEL motif or a transmembrane domain based on TMHMM (58).

Oomycetes other than *P. infestans* (Table S2) were searched for invertases using BLASTP and TBLASTN for protein and genome searches, respectively. Glycosylation was predicted using N-GlyDE and GlycoEP (59, 60).

10.1128/mBio.01251-20.10 TABLE S2 Sources of genome data. Download Table S2, PDF file, 0.05 MB. Copyright © 2020 Kagda et al. 2020 Kagda et al. This content is distributed under the terms of the Creative Commons Attribution 4.0 International license.

### Data availability.

RNA-seq data have been deposited in NCBI GEO under BioProject nos. PRJNA361417 and PRJNA407960.
