# Supplementary figures and images for: Invertases in Phytophthora infestans Localize to Haustoria and Are Programmed for Infection-Specific Expression
Source: mBio. 2020 Oct 13;11(5):e01251-20. doi: 10.1128/mBio.01251-20 (PMC7554665; doi:10.1128/mBio.01251-20)

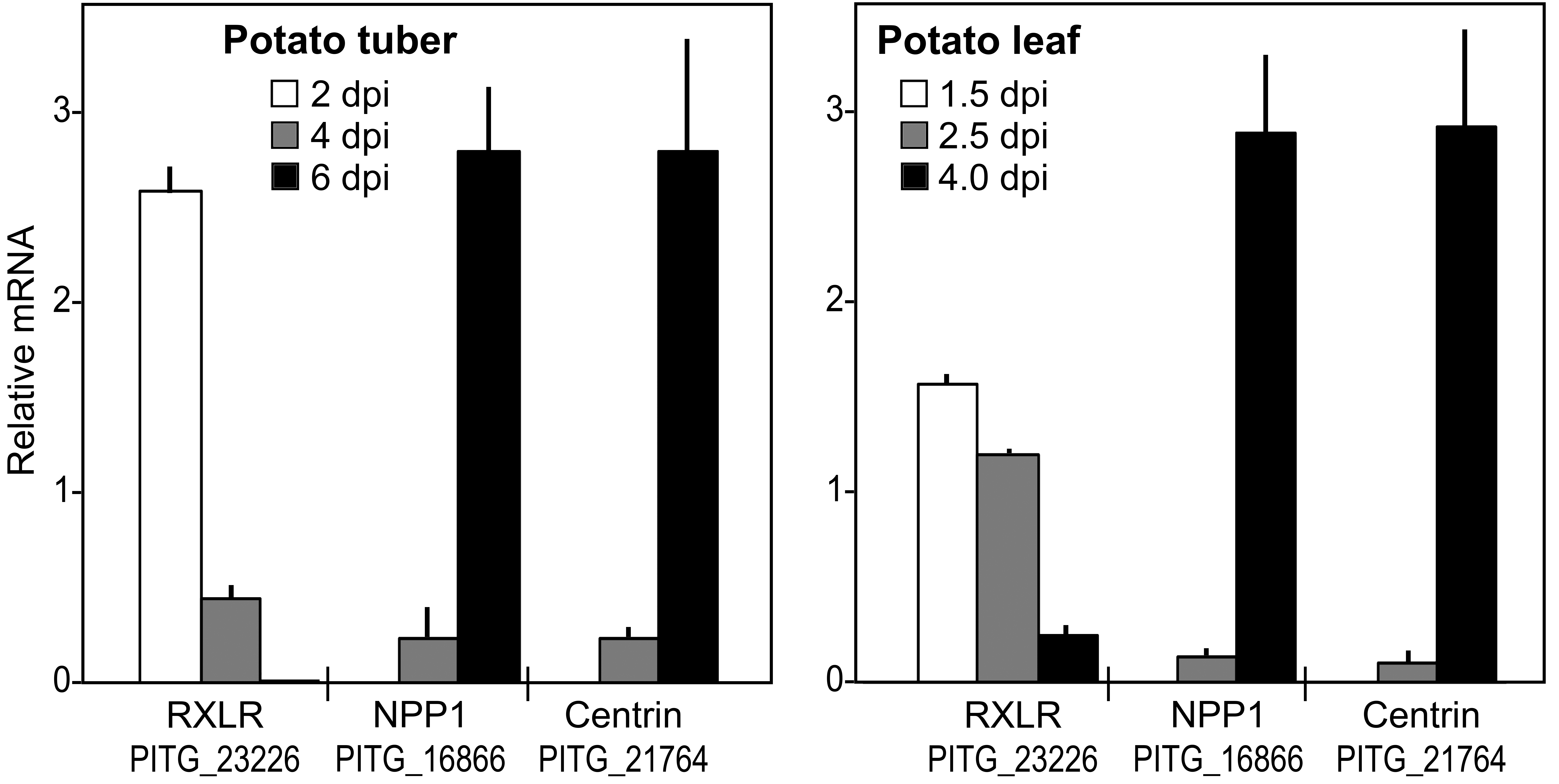

Supplement: FIG S1 [file mBio.01251-20-sf001.tif]

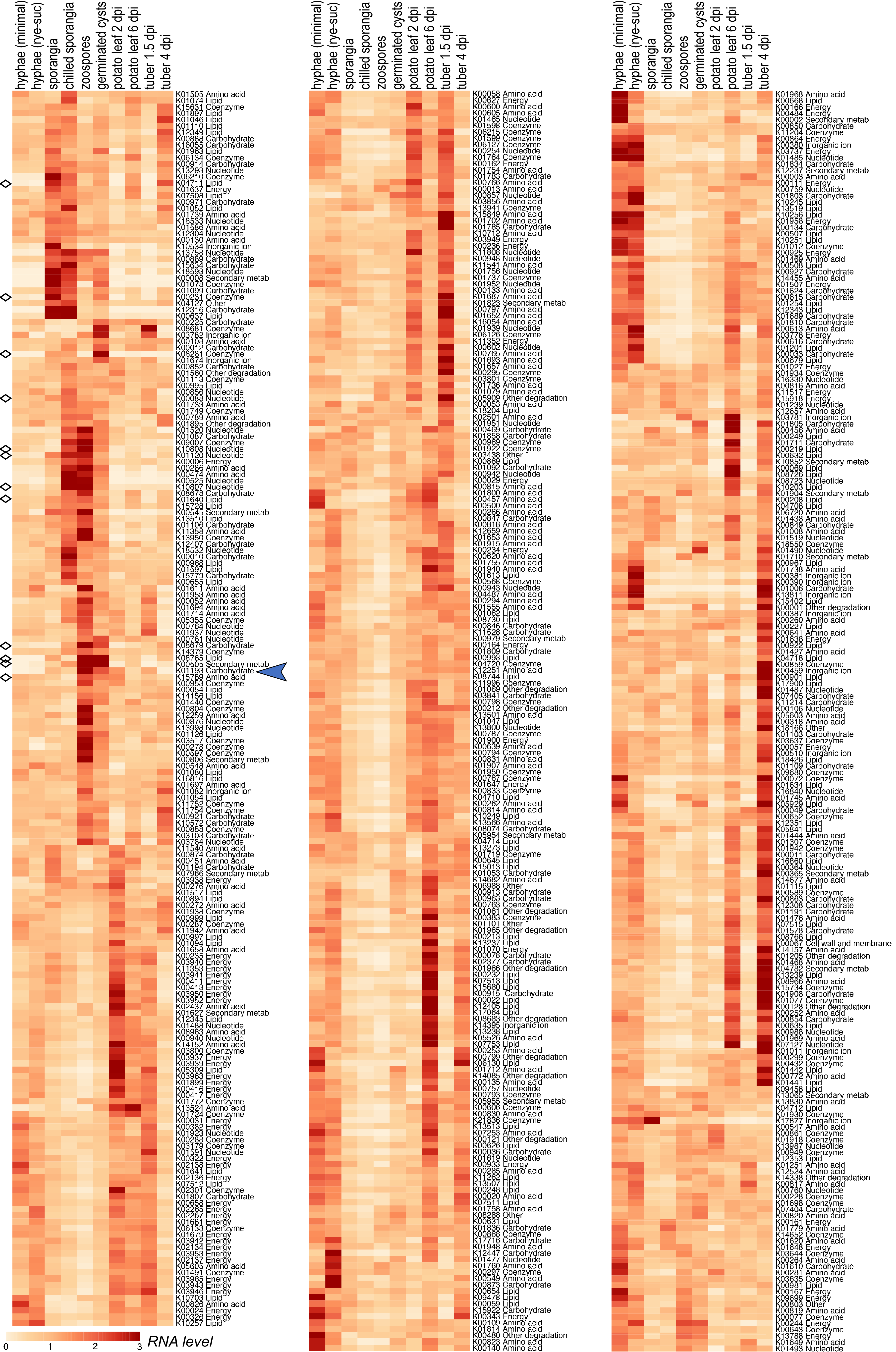

Supplement: FIG S2 [file mBio.01251-20-sf002.tif]

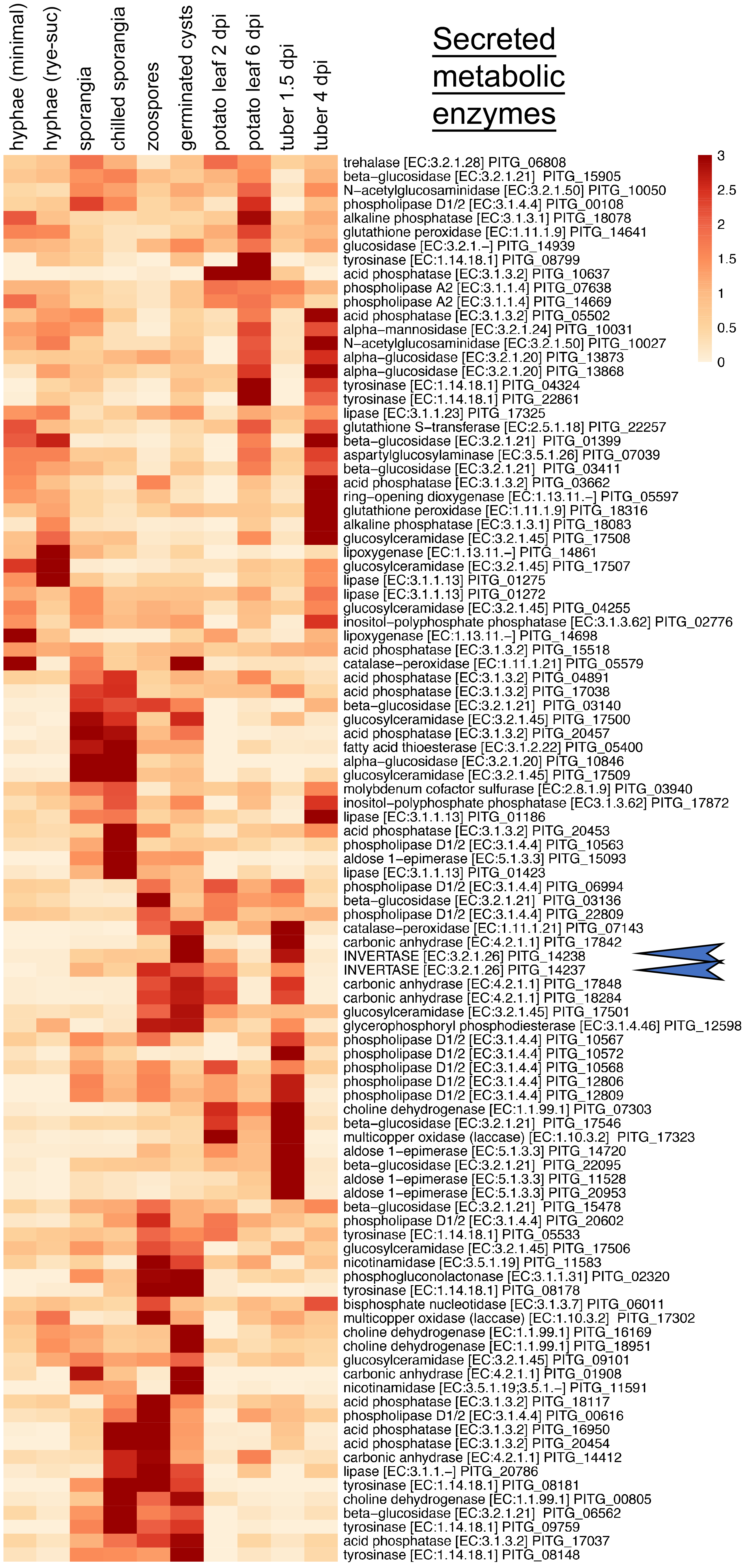

Supplement: FIG S3 [file mBio.01251-20-sf003.tif]

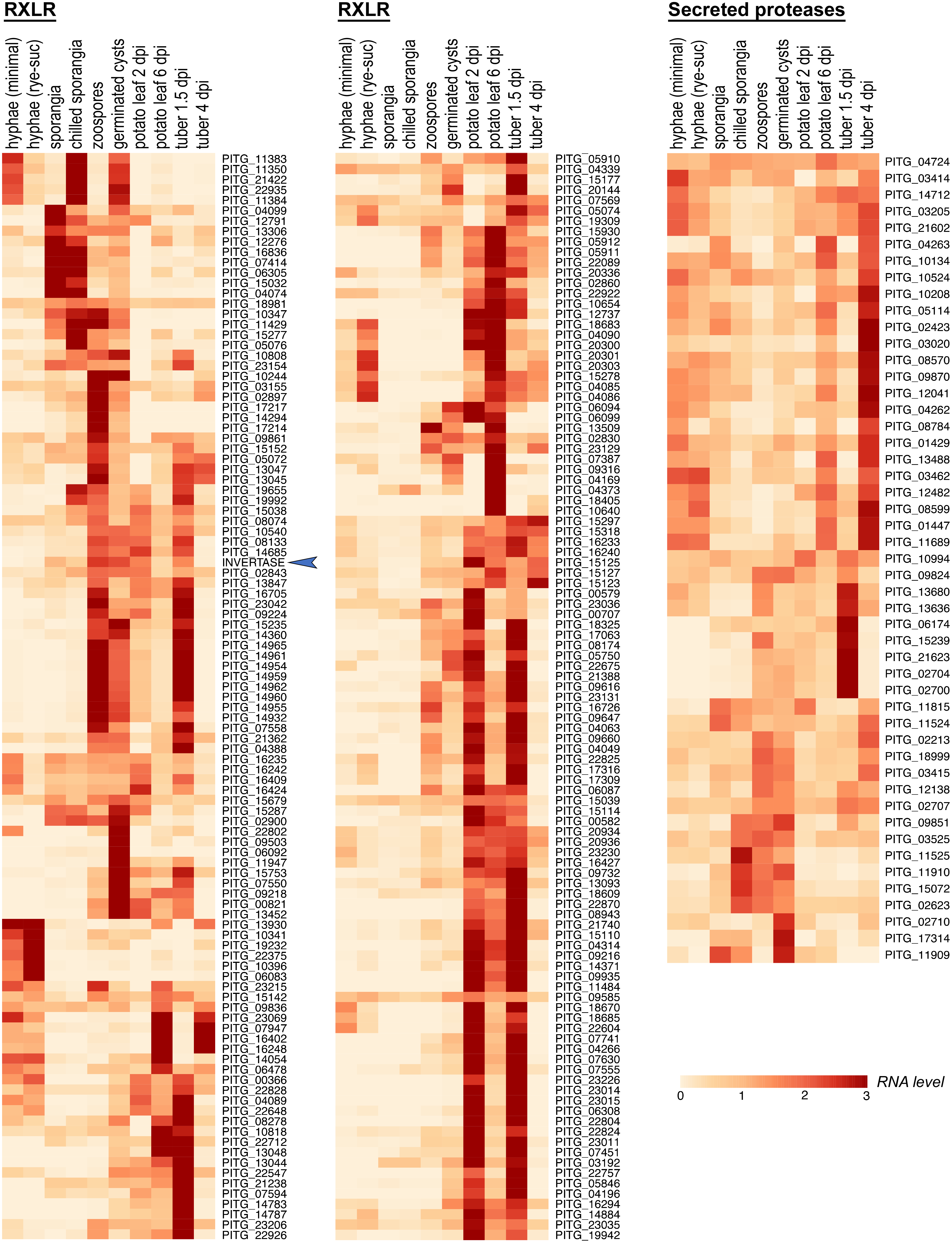

Supplement: FIG S4 [file mBio.01251-20-sf004.tif]

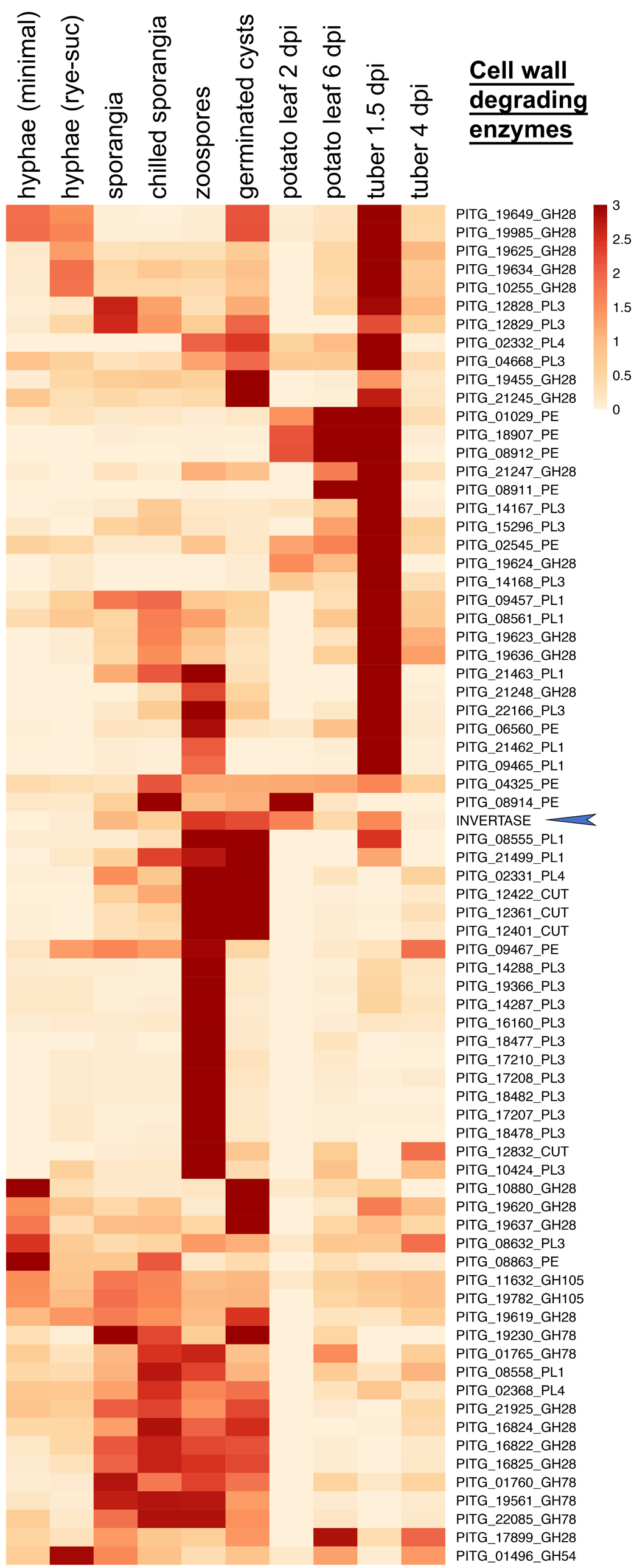

Supplement: FIG S5 [file mBio.01251-20-sf005.tif]

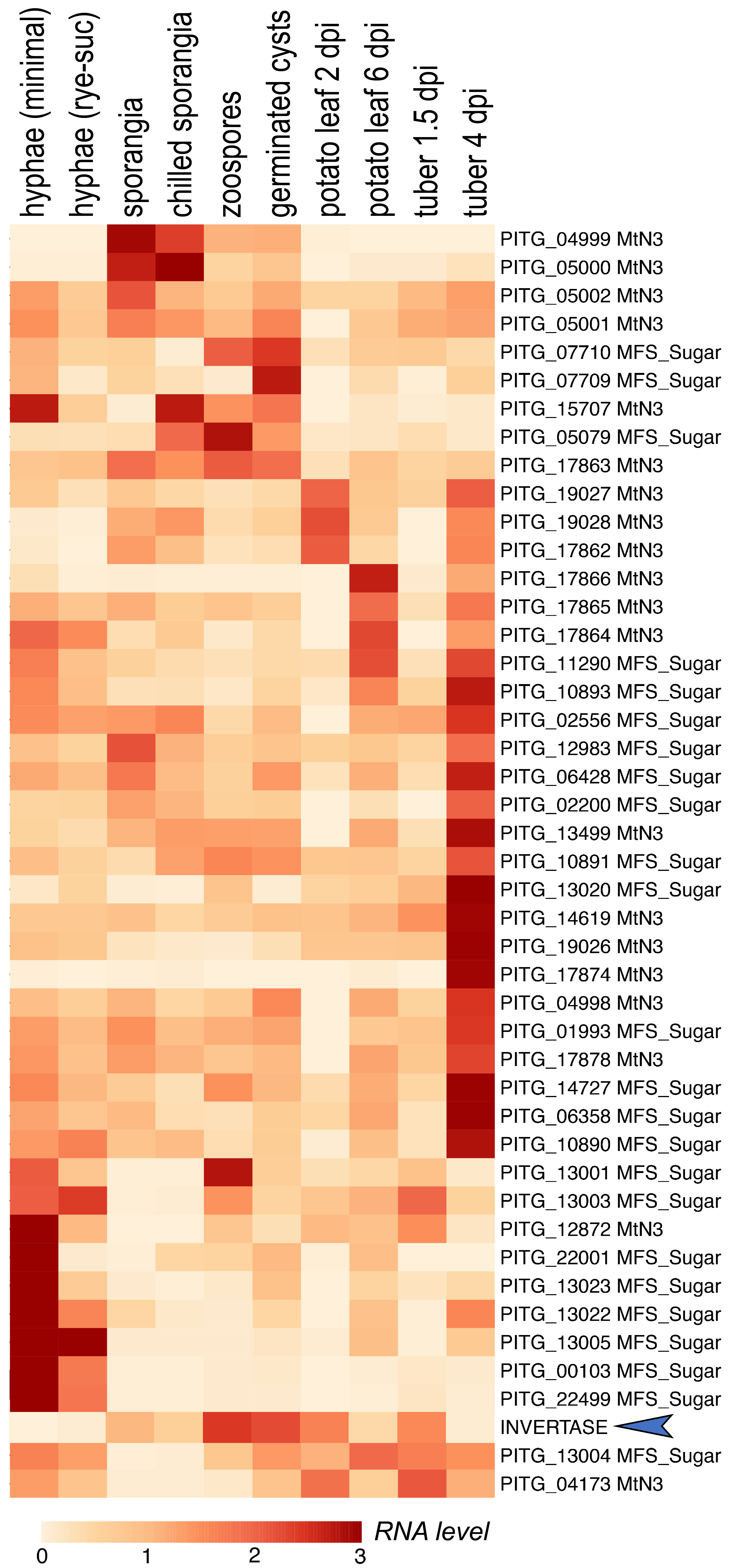

Supplement: FIG S6 [file mBio.01251-20-sf006.tif]

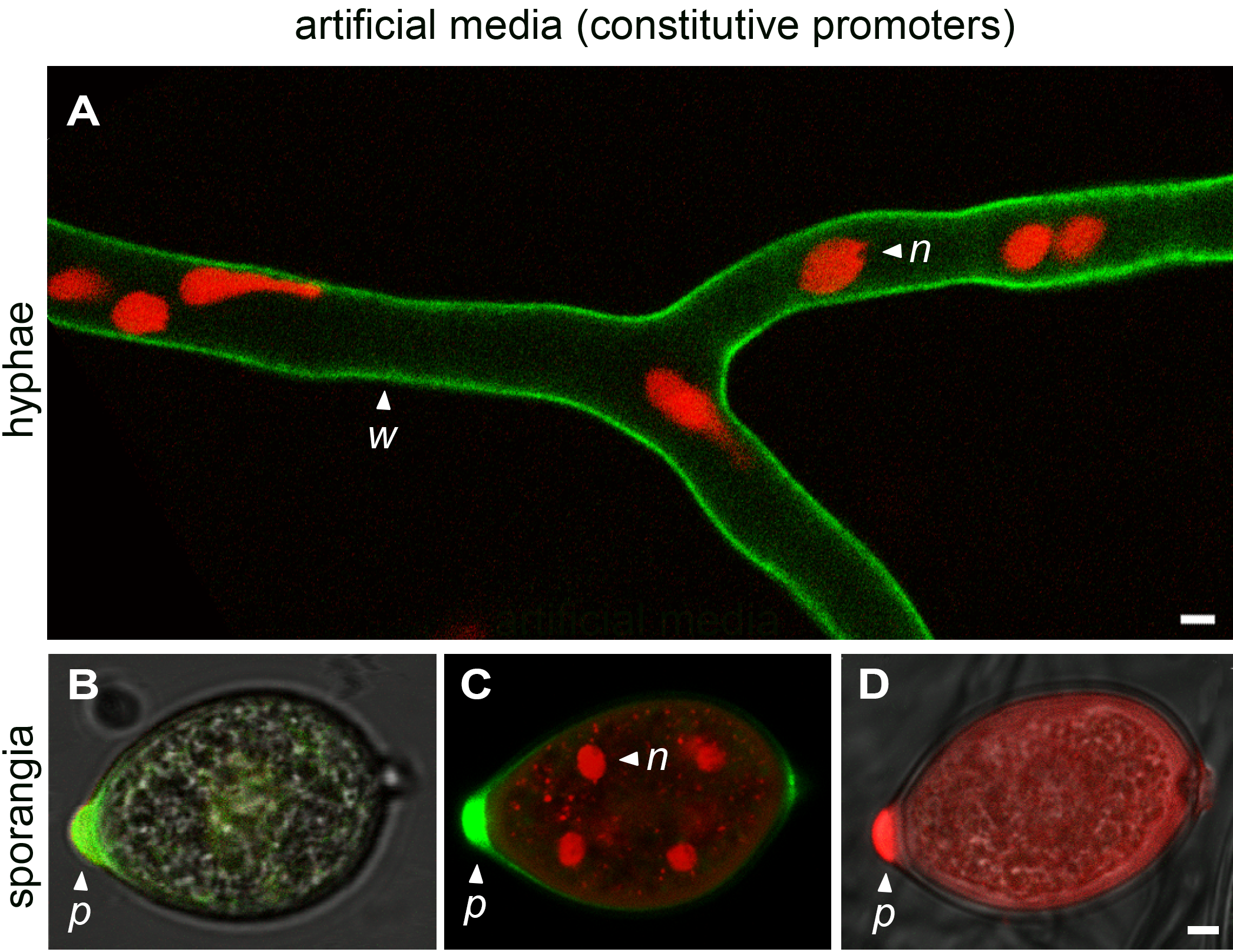

Supplement: FIG S7 [file mBio.01251-20-sf007.tif]
