# Supplementary material for: Invertases in Phytophthora infestans Localize to Haustoria and Are Programmed for Infection-Specific Expression
Source: mBio. 2020 Oct 13;11(5):e01251-20. doi: 10.1128/mBio.01251-20 (PMC7554665; doi:10.1128/mBio.01251-20)
Supplement: FIG S8 [file mBio.01251-20-sf008.pdf]

```
>PITG_12551 INF1
MNFRALFAATVAALVGSTSATTCTTSQQTVAYVALVSILSDTSFNQCSTDSGYSMLTATSLPTT

>PITG_00375 HMP1
MVLRAVRLIVQASLLLQVLQCGASVTGAQVNENFENTELTSNDKLGTVAPADIPSSQDENLKKQ

>PITG_14238 INVERTASE
MKVIPSIMAFSALVAALVFSPENAHAAIVEAEPGTELFEQFRPVYHFVAREKWMNDPCAPYYDE

>PITG_21410 INF4
MNFVALIAVTVAVLVGSTNAAACTAKQQTAAYNTLVSLLSEASFSTCSKDSGYSMITSKTLPRP

>PITG_22926 RXLR
MLRSFLLIVATVSLFGQCKPLPLATSPVSDAVRAPHRSTHETRFLRTNDEERGATMTLAGVLRD

>PITG_01029 Pectinesterase
MQIFAPLVALASLAAASEGACTGTNARTTPPPGAIVIDATGAYSGSFKTVSEGVANLPKTAVQQ
```

Fig. S8. N-terminal regions of proteins mentioned in this paper. Displayed are the first 64 amino acids of the INF1 (PITG_12251), HMP1 (PITG_00375), and invertase (PITG_14238) proteins. Grey shading indicates the signal peptides predicted by TargetP 2.0 (28). The regions used in tdTomato fusions involving INF1 and HMP1 are underlined. PITG_21410, PITG_22926, PITG_01029, and PITG_14371 are the secreted INF4, pectinesterase, and RXLR proteins shown in other studies to concentrate near haustoria (27). Sequences C-terminal of the signal peptide are dissimilar in chemical features such as hydropathy, ranging from moderately hydrophilic to hydrophobic, and lack conserved motifs based on alignments and motif discovery programs.
