# Supplementary material for: Invertases in Phytophthora infestans Localize to Haustoria and Are Programmed for Infection-Specific Expression
Source: mBio. 2020 Oct 13;11(5):e01251-20. doi: 10.1128/mBio.01251-20 (PMC7554665; doi:10.1128/mBio.01251-20)
Supplement: TABLE S2 [file mBio.01251-20-st002.pdf]

Table S2. Sources of genome data

Species	Source (Genbank unless specified otherwise)
Achlya hypogyna	GCA_002081595.1
Albugo candida	GCA_001078535.1
Albugo laibachii	GCA_902706625.1
Aphanomyces astaci	GCA_000520075.1
Aphanomyces invadans	GCA_000520115.1, GCA_003546525.1
Aphanomyces stellatus	GCA_009835185.1, GCA_900243725.1, GCA_900708865.1
Bremia lactucae	GCA_004359215.1
Hyaloperonospora arabidopsidis	GCA_000173235.2
Lagenidium giganteum	GCA_002286825.1
Nothophytophthora	GCA_001712635.2
Paralagenidium karlingii	GCA_002980415.1
Peronospora effusa	GCA_003843895.1
Phytophthora capsici	GCA_000325885.1
Phytophthora cinnamomi	Joint Genome Institute (CBS 144.22)
Phytophthora infestans	GCA_000142945.1 and (17)
Phytophthora palmivora	GCA_002911725.1
Phytophthora parasitica	GCA_000247585.2
Phytophthora ramorum	GCA_000149735.1
Phytophthora sojae	GCA_000149755.2
Phytopythium vexans	GCA_000387545.2, GCA_003413675.1
Pilasporangium sp.	GCA_001600495.1
Plasmopara halstedii	GCA_900000015.1
Plasmopara viticola	GCA_001695595.3
Pythium aphanidermatum	GCA_000387445.2
Pythium arrhenomanes	GCA_000387505.2
Pythium insidiosum	GCA_001029375.1, GCA_000764265.1
Pythium irregulare	GCA_000387425.2
Pythium iwayamai	GCA_000387465.2
Pythium oligandrum	GCA_005966545.1, GCA_001573145.1
Pythium splendens	GCA_006386115.1
Pythium ultimum	GCA_000143045.1
Salisapilia sapeloensis	PRJNA487262
Saprolegnia diclina	GCA_000281045.1
Saprolegnia parasitica	GCA_000151545.2
Thraustotheca clavata	GCA_002081575.1
